# Prevalence of Tobacco Smoking and Determinants of Success in Quitting Smoking among Patients with Chronic Diseases: A Cross-Sectional Study in Rural Western China

**DOI:** 10.3390/ijerph14020167

**Published:** 2017-02-09

**Authors:** Hang Fu, Da Feng, Shangfeng Tang, Zhifei He, Yuanxi Xiang, Tailai Wu, Ruoxi Wang, Tian Shao, Chunyan Liu, Piaopiao Shao, Zhanchun Feng

**Affiliations:** 1 School of Medicine and Health Management, Tongji Medical College, Huazhong University of Science & Technology, Wuhan 430074, China; fuhang@hust.edu.cn (H.F.); fengdahust@gmail.com (D.F.); sftang@hust.edu.cn (S.T.); ferry890520@gmail.com (Z.H.); wtailai2-c@my.cityu.edu.hk (T.W.); ruoxiwang@hust.edu.cn (R.W.); stian75309@hust.edu.cn (T.S.); lcy93@hust.edu.cn (C.L.); spp19930728@hust.edu.cn (P.S.); 2 School of Management, Hubei University of Chinese Medicine, Wuhan 430065, China; stephenhsiang@gmail.com

**Keywords:** tobacco control, chronic diseases, patients, quit smoking, western China, rural areas

## Abstract

Tobacco use is one of the behavioral risk factors for chronic diseases. The aim of the study was to investigate smoking prevalence in chronically ill residents and their smoking behavior in western rural China, to identify factors associated with success in quitting smoking, and to provide appropriate intervention strategies for tobacco control. Cross-sectional survey data from patients with chronic diseases from rural western China were analyzed. Among the 906 chronically ill patients, the current smoking prevalence was 26.2%. About 64.3% of smokers with chronic diseases attempted to quit smoking, 21.0% of which successfully quitted. The odds ratio (OR) of smokers with only one chronic disease to quit smoking successfully was higher than that of those who have other diseases (OR = 2.037, 95% confidence interval (CI) = 1.060–3.912; *p* < 0.05). The smokers who were always restricted to smoking in public places were more likely to quit smoking successfully than those who were free to smoke (OR = 2.188, 95% CI = 1.116–4.291; *p* < 0.05). This study suggests that health literacy, comorbidity of diseases, and psychological counseling should be considered when developing targeted tobacco prevention strategies. Strengthening tobacco control measures in public places such as rural medical institutions will be effective.

## 1. Introduction

The World Health Organization reported that approximately 1.3 billion people smoke, of which more than five million people die globally each year because of smoking. More than 80% of tobacco-attributable deaths are predicted to occur in developing countries [1]. China, as the largest developing country in the world, is also the largest producer and consumer of tobacco, accounting for approximately 40% of the global production and one-third of the global consumption [2]. Treatment of smoking-related diseases accounts for almost 6% of the total medical expenses in China [3].

Tobacco use is one of the behavioral risk factors for many chronic diseases, including cardiovascular diseases, diabetes, and inflammatory diseases [4,5]. One in six chronic disease deaths is caused by tobacco use [6]. Scholars have proposed effective interventions to reduce the prevalence of chronic diseases, of which tobacco control is identified as the most urgent and immediate priority [6,7]. The three main pillars of chronic disease prevention are healthy diet, physical activity, and avoidance of tobacco smoking [8]. On the one hand, the occurrence of chronic diseases can be prevented if people do not smoke. On the other hand, chronically ill residents who have been smokers can manage their disease well if they quit smoking successfully. However, smoking prevalence among residents with chronic diseases has not been properly documented [9].

Chronic diseases are becoming a serious public health concern in China [10]. The prevalence of chronic diseases is rapidly increasing in rural areas, especially in rural western China which is characterized by low economic status, inadequate health resources, and distinctive historical and cultural backgrounds [11,12]. Previous studies attributed the low health literacy and some health-related risk factors (tobacco use, drinking, lack of exercise, unreasonable dietary structure) as the important contributors to chronic diseases [13,14]. For example, rural residents in the northwest minority areas of China smoke heavily and consume alcohol excessively because of the cold living environment [15]. Considering that smoking behavior is one of the risk factors for chronic diseases, this study aims to investigate the smoking prevalence and behavior of smokers with chronic diseases in rural western China.

Studies reported the smoking prevalence in different populations, such as migrant workers [16,17] and medical students [18], in China and examined the determinants of tobacco smoking. However, minimal information is known about smoking prevalence in patients with chronic diseases and the factors affecting smoking behavior in rural China. Several studies also presented triggers for quitting smoking and factors associated with smoking cessation [19,20]. However, factors contributing to successfully quitting smoking among the chronically ill have yet to be characterized. This study aims to investigate smoking prevalence in chronically ill residents and their smoking behavior in western rural China. We also identified factors associated with success in quitting smoking to provide appropriate intervention strategies for tobacco control.

## 2. Methods

### 2.1. Study Design and Sample

Among chronic diseases, hypertension and diabetes are commonly associated with the tobacco use [21,22]. Therefore, the rural residents affected by these two chronic diseases were considered to be study population in this study. A cross-sectional survey in rural western China was conducted in April 2014. Rural residents with chronic diseases were recruited using a stratified multiple stage sampling method. Details of the sampling method are similar with those in our previous study [23]. Briefly, participants in rural areas from Qinghai, Xinjiang, and Inner Mongolia were randomly selected to ensure sample representation and determine the smoking prevalence in patients with chronic diseases. A total of 1080 patients who had registered for resident health records participated in the survey, and 906 of them provided valid data for the study. Face-to-face interviews were conducted by interviewers with each participant, and the aim and content of the survey were explained to all participants. All participants provided written informed consent. Approval for this study was obtained from the Ethics Committee of Tongji Medical College, Huazhong University of Science and Technology (IORG No: IORG0003571).

### 2.2. Data Collection and Measures

The following information were obtained from the participants: (1) socio-demographic characteristics including gender, age [24], number of people in household, educational level [23], and income [25]. Among them, number of people in household was divided into three parts: one to two, three to five, and more than 6, because a family that has three to five people is considered an average family size in China; (2) comorbidity of diseases [26], defined as a patient who suffered other diseases except for one chronic disease in this study, and obtained from patient declaration; (3) health literacy including health knowledge level and health behavior [27,28], exercise was measured by the question of “Do you take regular exercise?”, physical examination was measured by the question of “How often do you have a physical examination?”, and adherence to take medicine was measured by the question of “Do you take your medicine on time?”; (4) smoking-related conditions including family member smoker, which was measured by asking “Are there other family members smoking?”, average number of cigarettes smoked per day, quit attempts, and reasons in quitting smoking as well as restrictions in home, work place, and public places [16].

The participants were classified into four categories for analysis: (1) current smokers, defined as smoking within 30 days preceding the interview [29]; (2) failure quitters, defined as having tried to quit smoking previously but failed; (3) successful quitters, defined as having previously smoked and no longer smoke for at least 1 month before the survey; and (4) ever smokers, defined as having the experience of quitting smoking, including current smokers and successful quitters; (5) Never-smokers, defined as never smoking. To classify these categories we asked the following question: (1) “Have you smoked in the last 30 days?”; (2) “Have you ever tried to quit smoking?”; (3) “Have you succeeded in quitting smoking?”.

### 2.3. Statistical Analysis

All data were double-entered independently and validated using EpiData version 3.1 (Atlanta, GA, USA). All statistical analyses were performed using SPSS version 19.0 (SPSS Inc., Chicago, IL USA). All variables were presented as frequency distribution and percentage. Chi-square test was used to examine the associations of smoking prevalence with socio-demographic characteristics, health status, health knowledge and behavior, and use of health services. Smoking-related behavior and other variables among failure quitters and successful quitters were also tested by chi-square test. Only variables with statistically significant differences between failure and successful quitters were included in the binary logistic regression analysis model using conditional forward stepwise selection. Values with *p* < 0.05 (two-tailed) were considered statistically significant.

## 3. Results

### 3.1. Classification of Participants

**Figure 1:**
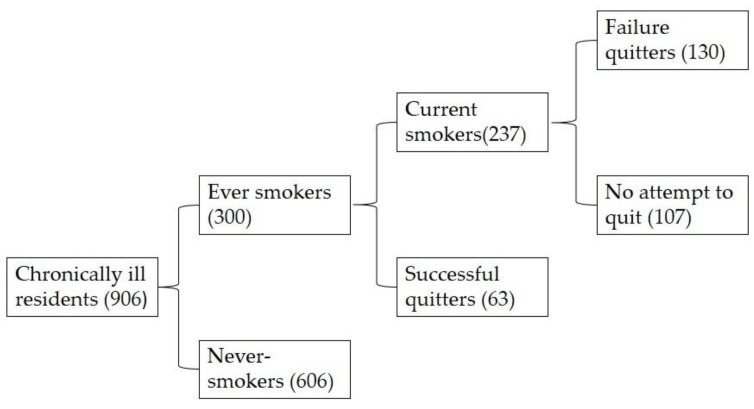
Chronically ill residents categorized by smoking conditions.

As shown in Figure 1, the number of “ever smokers” was 300 (male: 287; female: 13), accounting for 33.1% of the chronically ill patients. Among the 906 patients, 237 were classified as current smokers, indicating a 26.2% current smoking prevalence in our sample. In addition, a total of 193 participants (64.3% of ever smokers) attempted to quit smoking previously. Up to 130 of them had attempted to quit smoking in the past but were currently smoking, whereas 63 of them succeeded in quitting smoking. The success rate of smoking cessation was 21.0%.

### 3.2. Characteristics of the Study Population and Smoking Prevalence

Table 1 shows the current smoking prevalence categorized by demographics, health status, health knowledge, and behavior. Half of the participants (52.0%) were male, and the majority of the respondents (85.8%) were more than 50 years old. More than half of the respondents (51.9%) had three to five members in their households, and most of the families (73.3%) were living with an annual income below 30,000 RMB per year. More than half of the respondents (56.7%) had less than six years of education. More than half of the participants (53.1%) suffered other diseases in addition to chronic diseases. Although the education level of the participants was low, the health knowledge level of 54.6% of the participants was higher than that of the average population. The majority of the participants reported that they always exercise (62.0%), had a physical examination once a year (78.4%), took medicine on time (82.5%), and a received follow-up service four or more times a year (74.8%).

Table 1 shows that current smoking prevalence varied by various variables. The number of current smokers was significantly lower in female participants than in male participants (2.1% vs. 48.3%). Among the 237 current smokers, 77.7% had a relatively high education level. Participants who did not have a physical examination and did not take medicine on time were likely to smoke (*p* < 0.05).

### 3.3. Smoking Behavior and Reasons for Quitting Smoking

Table 2 shows the smoking behavior of the current smokers. Approximately half of the smokers reported other smokers in their family. Up to 35.4% current smokers consumed less than five cigarettes per day. More than half of them attempted to quit smoking previously (54.9%). Some current smokers reported that they experienced smoking restriction at home (47.7%), work (59.5%), and in public places (45.6%).

As shown in Table 3, common reasons for trying to quit smoking were having diseases (64.8%), doctor’s advice (18.1%), family member pressure (17.1%), economic factor (8.3%), and others (9.3%). No significant difference was observed in the reasons for smoking between those who failed and successful quitters.

### 3.4. Predictors Affecting Success in Quitting Smoking among Smokers with Chronic Diseases

Socio-demographic characteristics, health status, health knowledge and behavior, and smoking-related behavior were compared between failed and successful quitters. Gender, other diseases, exercise frequency, and smoking restriction at work and in public places were significantly associated with success in quitting smoking. As shown in Table 4, the successful quitters were more likely to be women, those not suffering other diseases, and those performing a small amount of exercise (not “always”). In addition, these successful quitters were restricted in smoking at work and in public places.

From Table 4, only five factors were significantly associated with success in quitting smoking, and these factors were included in the original model. A binary logistic regression analysis was then implemented to examine potential predictors of success in quitting smoking. The regression results are shown in Table 5. The odds ratio (OR) of smokers with chronic diseases who do not have comorbid diseases to quit smoking successfully was 2.037 times higher than that of those who have comorbid diseases (OR = 2.037, 95% confidence interval (CI) = 1.060–3.912; *p* < 0.05). The smokers who were always restricted from smoke in public places were more likely to quit smoking successfully than those who were free to smoke (OR = 2.188, 95% CI = 1.116–4.291; *p* < 0.05).

## 4. Discussion

Our study shows that the current smoking prevalence among chronically ill residents is 26.2%. Furthermore, 48.3% and 2.1% of the male and female patients were currently smoking cigarettes, respectively. The gender difference in smoking prevalence in our sample is similar to that reported in previous studies [31,32]. The current smoking prevalence among males in this study is lower than that of the national prevalence reported in the 2010 Global Adult Tobacco Survey (52.9%) [33]. Another study reported a higher smoking rate in the rural male population (66.8%) compared with urban residents (56.5%) [34]. However, in the present study, the number of current smokers with chronic diseases in rural areas is lower than that of the general population. This finding could be due to age structure and health status. In the present study, patients with chronic diseases surveyed were mostly middle-aged and elderly people because many young rural laborers move to urban areas to find jobs. These patients experienced multiple health issues. One study, which was designed to evaluate national-level tobacco control policies, reported the smoking rate of Chinese residents aged 55 and older was 33.4% [35].

Among chronically ill patients, those in rural areas with a relatively high educational level were likely to smoke. This result differs from a previous study that showed rural residents with a low educational level and those who engage in farm labor were likely to smoke [36]. The discrepancy could be due to the fact that residents with a high educational level have more demands for social interactions through cigarette smoking, which is known as a traditional Chinese gesture of goodwill. In addition, patients who did not take a physical examination and medicine on time were likely to smoke. This observation indicates that health literacy is a significant factor affecting smoking prevalence. Health literacy is composed of a complex set of skills, including making decisions that are helpful to health-related problem-solving [37]. Improved health literacy is associated with reductions in risk behaviors (including smoking behavior) for chronic diseases [38]. Therefore, health education and promotion programs should be delivered continuously and should focus on changes in health behavior.

Approximately 64.3% of ever smokers with chronic diseases attempted to quit smoking, 21.0% of which successfully quit. These two rates are higher than those reported in other studies targeting the general population; that is, approximately 20% to 25% of smokers attempted to quit but approximately half of them relapsed [31,39], whereas only 11% of smokers quit smoking successfully [33]. This result could be attributed to the fact that smokers with chronic diseases exhibited increased willingness to quit smoking and manifested more health concerns than ordinary people. We also found more than a third of current smokers with chronic diseases consume less than five cigarettes per day. It indicates that the degree of tobacco dependence of these smokers may not be very serious, and there are many potential possibilities to help them quit smoking. More research is needed into how to help the smokers with chronic diseases quit smoking by using tobacco-dependence treatment programs.

The primary reason for attempting to quit smoking among chronically ill patients is the effect on disease. Similarly, a previous study showed that health concerns are the foremost trigger for quitting or attempting to quit [19]. However, we found that the patients easily become failed quitters if they have other diseases in addition to one chronic disease. Hence, chronically ill patients will be inclined to refuse to stop smoking when they suffer from several diseases at the same time. Comorbidity of diseases is a significant factor in the quitting process. We can infer that smoking is regarded as a way to relieve pain and smokers exhibit a negative attitude toward the health effects of smoking cessation. Moreover, a doctor’s advice is the second reason for quitting or attempting to quit smoking. Intervention and smoking cessation advice offered by medical professionals significantly affect the smoking behavior of patients [40].

The regression model showed that smokers who were always restricted to smoking in public places are likely to quit smoking successfully. Although the state of existing tobacco control regulations in China and their enforcement remain at an early stage [41], the effectiveness of tobacco control regulations in public places is supported by the results of this study. Smoking restrictions in public places can raise awareness of the health risks of smoking. Such restrictions can encourage smokers to attempt to quit [42]. Therefore, the government needs to strengthen measures for controlling tobacco smoking in public places, especially in rural medical institutions. Current smokers with chronic diseases can improve their awareness about the health effects of smoking by accessing health services.

Although we tried to find the differences between failed and successful quitters among chronically ill patients, the missing distinction between daily and occasional smokers may introduce a bias in the comparative analyses. Participants who successfully stopped smoking daily and cut down to occasional smoking were denominated as failures, although they made a more qualified achievement and yielded greater health benefits than those “successful quitters” who just stopped occasional smoking. Therefore, future studies may further investigate the differences between daily and occasional smokers, and identify the factors associated with different stages or degrees of quitting smoking.

## 5. Limitations

This study presents several limitations. First, the category “current smokers” might cause unanticipated ambiguity in the smoking status subcategories, and the missing distinction between daily and occasional smokers might influence the disparities in other variables. Second, the variables for smokers in the questionnaire are incomplete, because the object of this survey was mainly to investigate the health knowledge and behavior of patients with chronic diseases, as well as their health services utilization. The smoking condition is only a part of the investigation, and some relevant questions were not included. Third, the survey results only included patients with chronic hypertension or diabetes, and other chronic diseases were not studied. Fourth, the multicollinearity of the independent variables was not checked. Moreover, the sample size of this study was small because of the low population density and the complex terrain. Lastly, the health knowledge level may suffer from information bias caused by the use of close-ended questions, which may have allowed patients to guess the correct answer.

## 6. Conclusions

Tobacco use is one of the behavioral risk factors for many chronic diseases. In this study, we determined smoking prevalence in chronically ill residents and identified factors associated with success in quitting smoking in western rural China. The study revealed that the current smoking prevalence in patients with chronic diseases is low when compared to the general population. Moreover, the success rate of smoking cessation is higher in the studied population than that in the general population. In addition, if smokers with chronic diseases do not have comorbidity of diseases or they are restricted to smoking in public places, these smokers are likely to quit smoking successfully. This study suggests that health literacy, comorbidity of diseases, and psychological counseling should be considered when developing targeted tobacco prevention strategies. The government also needs to strengthen tobacco control measures in public places, especially in rural medical institutions.

## Figures and Tables

**Table 1:** Current smoking prevalence categorized by demographics, health status, health knowledge, and behavior.

Variable	Total (*N*)	Current Smokers		χ^2^	*p*
		*N*	%		
Gender				250.183	<0.001
Male	472	228	48.3		
Female	434	9	2.1		
Age				1.866	0.389
<50	129	39	30.2		
50–65	370	99	26.8		
>65	407	99	24.3		
Number of people in household				5.414	0.067
1–2	97	34	35.1		
3–5	470	124	26.4		
>6	339	79	23.3		
Educational level				60.390	<0.001
Less than 6 years study	512	83	16.2		
6–9 years study	271	107	39.5		
Over 9 years study	123	47	38.2		
Annual disposable household income *				0.383	0.658
<RMB10,000	242	68	28.1		
RMB10,000–RMB29,999	422	105	24.9		
>RMB30,000	242	64	26.4		
Comorbidity of diseases				1.932	0.165
Yes	481	135	28.1		
No	425	102	24.0		
Health knowledge level				1.661	0.198
Below	411	116	28.2		
Higher	495	121	24.4		
Exercise				2.444	0.295
Always	562	157	27.9		
Sometime	221	52	23.5		
Hardly	123	28	22.8		
Frequency of physical examination				10.413	0.005
Once a year	710	170	23.9		
Occasion	110	42	38.2		
Hardly	86	25	29.1		
Take medicine on time				11.966	0.001
Yes	747	178	23.8		
No	159	59	37.1		
Total	906	237	26.2		

Note: * Average disposable household income in 2014 for rural families is 49,497 RMB, and for urban families is 88,683 RMB [30]. The mean of annual disposable household income in our sample regions is 22,061 RMB.

**Table 2:** Smoking conditions among current smokers.

Variable	*N*	%
Family member smoker		
Yes	113	47.7
No	124	52.3
Average number of cigarettes smoked per day		
<5	84	35.4
5–10	59	24.9
11–20	60	25.3
>20	34	14.3
Quit attempts previously		
Yes (=failure quitters)	130	54.9
No	107	45.1
Smoking restrictions in home		
Yes	113	47.7
No	124	52.3
Smoking restrictions in work place		
Yes	141	59.5
No	96	40.5
Smoking restrictions in public place		
Yes	108	45.6
No	129	54.4

**Table 3:** Reasons for trying to quit smoking among failed and successful quitters.

Reasons	Total *N* (%)	Failure Quitters *N* (%)	Successful Quitters *N* (%)	χ^2^	*p*
Disease effect	125 (64.8)	85 (65.4)	40 (63.5)	0.067	0.796
Doctor’s advice	35 (18.1)	28 (21.5)	7 (11.1)	3.108	0.078
Family member pressure	33 (17.1)	26 (20.0)	7 (11.1)	2.365	0.124
Economic factor	16 (8.3)	9 (6.9)	7 (11.1)	0.979	0.322
Others	18 (9.3)	10 (7.7)	8 (12.7)	1.258	0.262

**Table 4:** Factors significantly associated with success in quitting smoking.

Variable	Failure Quitters *N* (%)	Successful Quitters *N* (%)	χ^2^	*p*
Gender			-	0.011 ^a^
Male	130 (100.0)	59 (93.7)		
Female	0 (0.0)	4 (6.3)		
Comorbidity of diseases			7.593	0.006
Yes	79 (60.8)	25 (39.7)		
No	51 (39.2)	38 (60.3)		
Exercise			7.632	0.022
Always	85 (65.4)	29 (46.0)		
Sometime	27 (20.8)	24 (38.1)		
Never	18 (13.8)	10 (15.9)		
Smoking restrictions in work place			10.099	0.001
Yes	45 (34.6)	37 (58.7)		
No	85 (65.4)	26 (41.3)		
Smoking restrictions in public place			10.293	0.001
Yes	61 (46.9)	45 (71.4)		
No	69 (53.1)	18 (28.6)		

^a^ Fisher exact test of probabilities.

**Table 5:** Outcome of logistic regression analysis for examining predictors correlated with success in quitting smoking.

Predictors	Reference	B	*p*	OR	95% CI	
					Lower	Upper
Gender	Female	−21.708	0.999	0.000	0.000	0.000
Comorbidity of diseases	Yes	0.711	0.033	2.037	1.060	3.912
Smoking restrictions in public place	No	0.783	0.023	2.188	1.116	4.291
Constants		20.103	0.999	5.377		
